# The Hartung-Knapp-Sidik-Jonkman method for random effects meta-analysis is straightforward and considerably outperforms the standard DerSimonian-Laird method

**DOI:** 10.1186/1471-2288-14-25

**Published:** 2014-02-18

**Authors:** Joanna IntHout, John PA Ioannidis, George F Borm

**Affiliations:** 1 Department for Health Evidence (HEV), Radboud University Medical Center, Huispost 133, P.O. box 9101, Nijmegen, HB 6500, The Netherlands; 2 Stanford Prevention Research Center, Department of Medicine, Stanford University School of Humanities and Sciences, Stanford, CA 94305, USA; 3 Department of Health Research and Policy, Stanford University School of Medicine, Stanford, CA 94305, USA; 4 Department of Statistics, Stanford University School of Humanities and Sciences, Stanford, CA 94305, USA

**Keywords:** Meta-analysis, Clinical trial, Trial size, Heterogeneity, Type I error, Random effects, Cochrane Database of Systematic Reviews

## Abstract

**Background:**

The DerSimonian and Laird approach (DL) is widely used for random effects meta-analysis, but this often results in inappropriate type I error rates. The method described by Hartung, Knapp, Sidik and Jonkman (HKSJ) is known to perform better when trials of similar size are combined. However evidence in realistic situations, where one trial might be much larger than the other trials, is lacking. We aimed to evaluate the relative performance of the DL and HKSJ methods when studies of different sizes are combined and to develop a simple method to convert DL results to HKSJ results.

**Methods:**

We evaluated the performance of the HKSJ versus DL approach in simulated meta-analyses of 2–20 trials with varying sample sizes and between-study heterogeneity, and allowing trials to have various sizes, e.g. 25% of the trials being 10-times larger than the smaller trials. We also compared the number of “positive” (statistically significant at p < 0.05) findings using empirical data of recent meta-analyses with > = 3 studies of interventions from the Cochrane Database of Systematic Reviews.

**Results:**

The simulations showed that the HKSJ method consistently resulted in more adequate error rates than the DL method. When the significance level was 5%, the HKSJ error rates at most doubled, whereas for DL they could be over 30%. DL, and, far less so, HKSJ had more inflated error rates when the combined studies had unequal sizes and between-study heterogeneity. The empirical data from 689 meta-analyses showed that 25.1% of the significant findings for the DL method were non-significant with the HKSJ method. DL results can be easily converted into HKSJ results.

**Conclusions:**

Our simulations showed that the HKSJ method consistently results in more adequate error rates than the DL method, especially when the number of studies is small, and can easily be applied routinely in meta-analyses. Even with the HKSJ method, extra caution is needed when there are = <5 studies of very unequal sizes.

## Background

The commonly used method for a random effects meta-analysis is the DerSimonian and Laird approach (DL method) [1]. It is used by popular statistical programs for meta-analysis, such as Review Manager (RevMan [2]) and Comprehensive Meta-analysis [3]. However, it is well known that the method is suboptimal and may lead to too many statistically significant results when the number of studies is small and there is moderate or substantial heterogeneity [4-10]. If a treatment is inefficacious and testing is done at a significance level of 0.05, the error rate should be 5%, i.e. only one in 20 tests should result in a statistically significant result. For the DL method, the error rate can be substantially higher, unless the number of studies is large (≫ 20) and there is no or only minimal heterogeneity [4-10].

Given this deficiency, alternative methods for random effects meta-analysis have been proposed. In particular, the method described by Hartung and Knapp [4-6] and by Sidik and Jonkman [11,12] (HKSJ method) is claimed to be simple and robust [13]. Simulations have shown that the HKSJ method performs better than DL, especially when there is heterogeneity and the number of studies in the meta-analysis is small [4-14]. This means that for most meta-analyses the HKSJ method might be more appropriate than the conventional DL method. In a sample of 22453 meta-analyses, Davey et al. show that the number of studies in a meta-analysis is often relatively small, with a median of 3 studies (Q1-Q3: 2–6), and only 1% of meta-analyses containing 28 studies or more [15]. Some detectable heterogeneity is present in about half of meta-analyses of clinical studies [15-18].

Based on earlier results that showed that the results of a single large trial were unreliable [19], we hypothesized that the meta-analyses methods, including HKSJ, would perform less adequately when the meta-analysis is carried out on a mixture of very unequal-sized studies, e.g. one large and several small trials. Such a situation is not uncommon. In a random sample of 186 systematic reviews of the Cochrane Database [18] the ratio between large and small trial sizes ranged between 1 and 1650, with a median of 5 and an interquartile range from 3 to 10. Sixty per cent of the reviews contained no large trials, but 40% had one trial that was at least twice as large as the median trial size, 25% had one trial that was at least five times larger, and 10% had one trial that was even 10 times larger.

Although several simulations have shown that the HKSJ method performs better than the DL method, the focus in these studies was not on a systematic evaluation of the effects of specific trial size mixtures in combination with low trial numbers. They either only reported the overall results of various mixtures combined or they studied only a limited number of combinations. In order to investigate the impact of unequal study sizes, we used simulations, mimicking such realistic conditions rather than situations where trials have implausibly similar sample sizes. We focused on meta-analyses with small numbers of studies (up to 20) with a dichotomous outcome (odds ratio, relative risk) or a continuous outcome. To mimic the variation in trial sizes, we explicitly varied the sample sizes of the trials within the simulated meta-analyses, varying from scenarios where all trials in a meta-analysis were of equal size, to scenarios with only one large trial, 10 times as large as the other trials, or one small trial, 10 times smaller than the other trials.

In order to complement the simulations, empirical data, based on recent meta-analyses - added or updated in 2012 - from the Cochrane Database of Systematic Reviews (CDSR) of interventions were used to assess the number of nominally statistically significant findings (with p < 0.05) of both methods in practice. This allows to examine whether inferences would be very different based on these two models.

Currently not all standard software packages like Review Manager provide an option to perform an HKSJ analysis, although the HKSJ method is computationally not complicated and the importance of suitable methods for meta-analyses with small numbers of trials is apparent. Version 3.0 of Comprehensive Meta-analysis [3] will contain the HKSJ method (personal communication by Julio Sánchez-Meca, September 2013). Also the R package metafor [20] and the metareg command in Stata [21] include the HKSJ method. However, not everybody will be acquainted with the use of R or Stata. Moreover, use of these packages is not straightforward when a post-hoc conversion is desired, i.e. when the results of a DL random effects analysis must be converted to the HKSJ approach. In order to fill this gap, we show step by step how the HKSJ analysis can be performed without the use of these packages, when the results of a common random effects (DL) meta-analysis are available, e.g. from a systematic review. This conversion is applicable for continuous outcomes and for outcomes where metrics are log-transformed, like the risk ratio (RR), odds ratio (OR), hazard ratio (HR) or Poisson rate. This simple modification of the common random effects analysis will improve the summary results, and it can be done through some basic calculations or a few statements in Excel. An Excel file is available as Additional file 1 web material. R code for the metafor package is provided in Appendix 3.

The simulations, the selection of empirical data and the statistical analysis are described in the Methods section. In the Results section the error rates for the DL and HKSJ methods for several realistic simulated scenarios are provided. For the Cochrane meta-analyses, we present the number of nominally statistically significant findings with the DL and HKSJ methods. The conversion of DL results into HKSJ results is illustrated, including examples from systematic reviews as presented in the Cochrane Library.

## Methods

We used simulated data as well as empirical data of the Cochrane 2012 Issues to evaluate the DL and HKSJ approaches. The pooled effect estimate is equal for both approaches, but the methods differ with respect to the calculation of the confidence interval and the statistical test. For DL, these are based on the normal distribution, whereas for the HKSJ method, they are based on the t-distribution with the degrees of freedom equal to the number of trials minus one, and a weighted version of the DL standard error. Detailed statistical methods are presented in Appendix 1.

### Methods - simulations

Our first aim was to investigate the error rates of the HKSJ meta-analysis method in comparison to the common (DL) method for various realistic scenarios, i.e. combinations of study sizes, study size mixtures and heterogeneity in series of just a few trials. Therefore we simulated series of trials with two up to 20 studies, where each series provided the data for one meta-analysis. First, we considered series that consisted of equally sized trials, each with two groups of 25, 50, 100, 250, 500 or 1000 subjects. Second, we looked into series of trials with different trial sizes, i.e. the percentage of large trials was 25%, 50% or 75%, e.g. a series of one large trial and three small trials. Average group sizes were 100, 250, 500 or 1000 subjects, and the large trials had 10 times more subjects than the small trials. For example, a series of six small (normal) and two large trials, with an average group size of 100, has group sizes of 31 and 308 in the small and large trials, respectively. Third, we simulated extreme scenarios, in which a series had only small trials, except for one large one, or only large trials, except for one small one. Both continuous and dichotomous outcomes were evaluated. For continuous outcomes, a normally distributed overall mean difference between the group means was simulated. In the trials with a dichotomous outcome, the event rates in the groups varied between scenarios and ranged from 0.1 to 0.9, in steps of 0.2. The heterogeneity was superimposed and set at I^2^ = 0, 0.25, 0.50, 0.75 and 0.9. I^2^ represents the heterogeneity, i.e. the degree of inconsistency in the studies’ results, in comparison to the total amount of variation [16,22]. The levels correspond to no, low, moderate, high and very high heterogeneity, respectively [16].

Our aim was to evaluate the error rate, i.e. the percentage of statistically significant meta-analyses when the overall mean treatment difference was zero. Hence we simulated series with an overall treatment difference equal to zero and performed on each series a DL [1] and an HKSJ [11] random effects meta-analysis. The two-sided significance level was 0.05. For each scenario, we simulated 10,000 series of trials. In the ideal situation, 5% of the 10,000 meta-analyses should have a statistically significant result when the significance level is 0.05. For the scenarios with the dichotomous outcome we determined the error rate when the OR was evaluated (logistic model) and when the RR was estimated. In these cases, meta-analysis was done on the logarithmic scale, and the error rates were determined for OR = 1 or RR = 1. More details can be found in Appendices 1 and 2.

### Methods - empirical data from the 2012 Cochrane Database of Systematic Reviews

Cochrane Reviews are systematic reviews of primary research in human health care and health policy, and are internationally recognised as the highest standard in evidence-based health care [23]. The aim of the Cochrane collaboration is to provide accessible and credible evidence to guide decision making in medicine and public health. We were very fortunate that the UK Cochrane Editorial Unit provided us with the statistical data added to the CDSR in 2012, which allowed us to assess the number of statistically significant results in real data.

Many Cochrane reviews include multiple meta-analyses. Many of those overlap or are based on correlated data. Usually, the first analysis is the primary analysis. Hence, we decided to use per review only the first meta-analysis that was based on at least three studies. In order to maximize the number of meta-analyses, we used both the first continuous and the first binary outcome meta-analysis, whenever possible. Thus some systematic reviews provided none, and some provided one or two meta-analyses for our research. We always performed a random effects meta-analysis, even when the authors originally performed a fixed-effects analysis. Details can be found in Appendix 1.

It is impossible to determine which of the Cochrane reviews compared treatments that truly had equal efficacy. It is thus unknown which of the statistically significant results were in fact false positive findings, so we could not determine the false positive error rate. Hence we decided to present the total number of significant findings of the DL and HKSJ methods instead of the error rates. This provides an indication of the impact a change from DL to HKSJ would have in practice.

## Results

### Error rates for continuous outcomes

**Figure 1:**
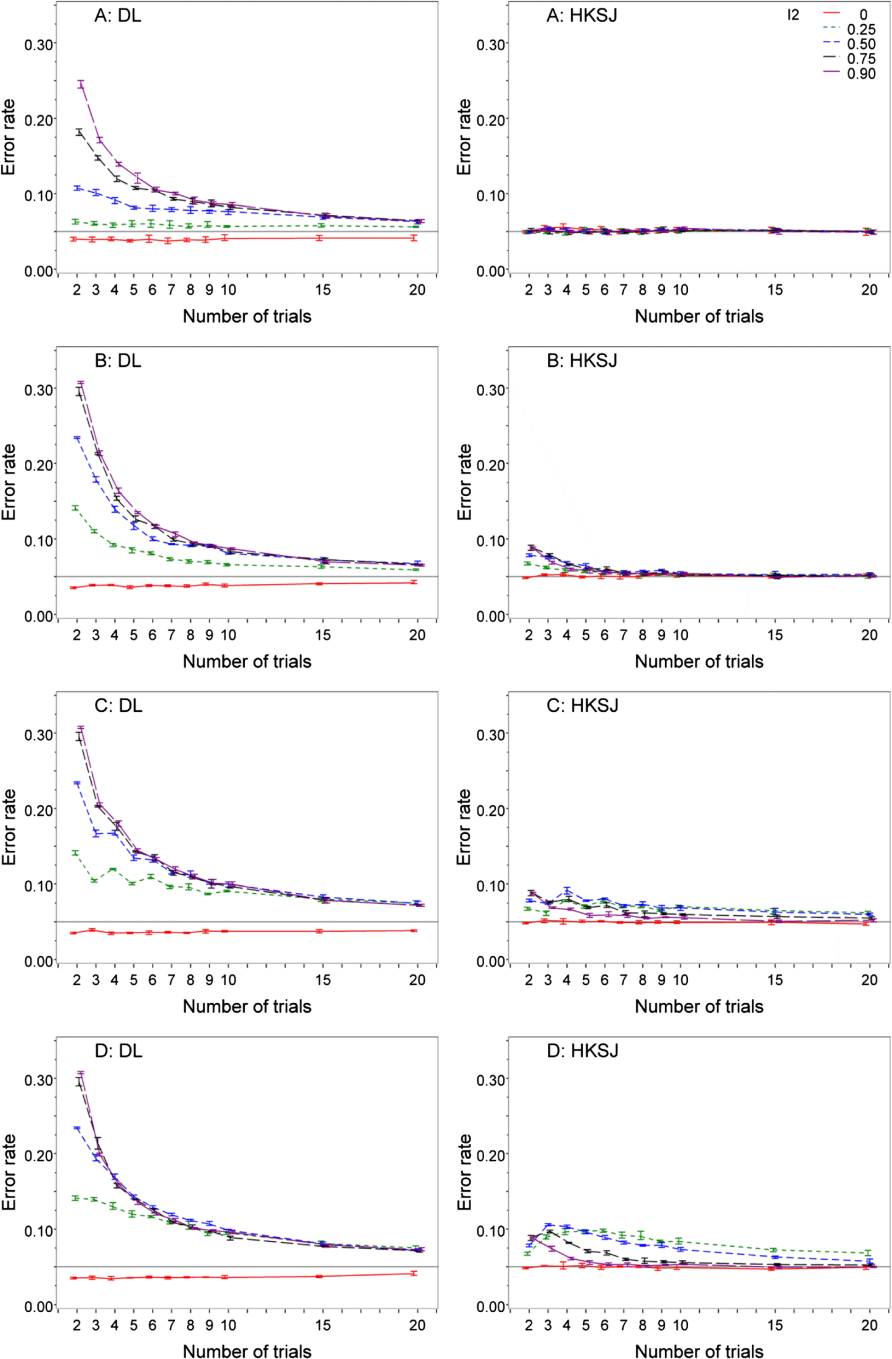
**DerSimonian-Laird and Hartung-Knapp-Sidik-Jonkman error rates for continuous outcomes, for various I**^**2 **^**and mixtures of trial sizes.** Legend: **A**: Equally sized trials; **B**: One small trial, 1/10th of other trials; **C**: 50–50 small and large trials (ratio 1:10); **D**: one large trial (10 times larger than other trials). Vertical bars refer to the minimum and maximum error rates over the group sizes. The lines connect the means of these error rates. DL: DerSimonian-Laird meta-analysis method. HKSJ: Hartung-Knapp-Sidik-Jonkman meta-analysis method.

The left side of Figure 1 shows the error rates for the DL method for the simulated mixtures of trial sizes. In general with unequal-sized trials, the type I error of DL was substantially inflated even with minimal heterogeneity, while with equal-sized trials minimal or modest heterogeneity did not inflate the type I error substantially. Figure 1A shows the error rates for a setting with studies of equal size, Figure 1B for one small trial, Figure 1C for equal numbers of large and small trials, and Figure 1D for a setting with one large trial, 10 times as large as the other trials. The heterogeneity levels are I^2^ = 0, 0.25, 0.5, 0.75 and 0.9, and the average study group sizes range between 25 and 1000. Vertical bars refer to the minimum and maximum error rates over the group sizes. The lines connect the means of these error rates. The error rates should all have been 5% (0.05), but for I^2^ ≥ 0.25, DL error rates were too large, even for series of 20 trials. For example, DL error rates for meta-analyses of five studies ranged between 5.7% for equally sized trials and 14.7% for mixtures of trial sizes (Table 1). In contrast, the error rates were too low (about 3-4%) when the I^2^ was 0. DL results for other, less extreme, mixtures of trial sizes were in between the results shown.In Figure 1 on the right side results for the HKSJ approach are presented. For equal trial sizes, the error rates of the HKSJ method were very appropriate. When the series contained only one small trial, the HKSJ error rates were approximately correct if the series consisted of more than five studies (Figure 1B). For series containing fewer trials, the error rates were higher, but not as high as the respective DL values. They were also too high when the percentage of small trials increased (Figure 1C). When there was only one large trial, the HKSJ error rates sometimes almost doubled (Figure 1D). When there was no heterogeneity, HKSJ error rates were roughly 5%. As expected, the group sizes had no impact on the error rates.Figure 1 shows that the HKSJ method always outperformed the common random effects DL method. The HKSJ error rate was usually roughly 5%. However, some mixtures of sizes, especially when there is only one large trial, lead to a doubling of the error rate to 10%. This occurred especially when heterogeneity was only moderate.

**Table 1:** Minimum and maximum error rates of DerSimonian-Laird and Hartung-Knapp-Sidik-Jonkman methods for mixtures of trial sizes

**Outcome**		**No of**	**Equally sized**		**One small trial**		**50–50**		**One large trial**	
	**I**^**2**^	**trials**	**DL**	**HKSJ**	**DL**	**HKSJ**	**DL**	**HKSJ**	**DL**	**HKSJ**
Continuous	0	2-20	3.4–4.6	4.5–6	3.4–4.5	4.7–5.4	3.3–4.1	4.6–5.4	3.2–4.4	4.5–5.7
0.25–0.9		2	6–25	4.7–5.4	13.8–30.9	6.5–9.2	13.8–30.9	6.5–9.2	13.8–30.9	6.5–9.2
		3	5.9–17.5	4.7–5.6	10.8–21.7	6–8	10.2–20.8	5.9–7.7	13.7–22.1	7.1–10.7
		4	5.6–14.2	4.5–5.5	9–16.8	5.6–7	11.9–18.4	6.6–9.6	12.6–17.3	5.9–10.5
		5	5.7–12.7	4.7–5.5	8.2–13.6	5.5–6.7	9.9–14.7	5.6–7.9	11.6–14.5	5.3–9.9
		10	5.6–8.8	4.8–5.6	6.4–8.8	5–5.6	9–10.3	5.4–7.2	8.5–10	5.3–8.8
		20	5.6–6.6	4.6–5.3	5.8–7.1	4.8–5.5	7.1–7.8	5–6.4	6.9–7.8	4.9–7.2
Risk ratio	0	2-20	0.9–4.2	2.1–6.9	2.8–4.1	2.7–6.5	3.0–4.1	2.7–6.8	2.7–4.3	2.7–5.5
0.25–0.9		2	2.5–26.3	2.8–6.7	14.3–33.7	6–10.2	14.3–33.7	6–10.2	14.3–33.7	6–10.2
		5	2.5–12.9	3.9–5.7	7.9–15	5.5–7.2	9.8–15.7	5.2–7.9	11.4–14.2	5.2–10.6
		10	2.6–8.9	2.7–5.4	6–9.7	3.8–5.7	8.6–11	4.8–9.1	7.3–10.1	3.6–8.7
Odds ratio	0	2-20	1.3–4.3	2.7–6.1	3–4	3–6.7	3–4	3–6.7	2.9–4.1	3–5.4
0.25–0.9		2	2.9–25.3	3.2–5.9	13.7–33.5	6.1–9.6	13.7–33.5	6.1–9.6	13.7–33.5	6.1–9.6
		5	3–12.7	3.9–5.3	7.9–14.4	5.4–6.9	9.9–15.8	5.3–8.1	11.6–14.2	5.2–10.5
		10	2.9–8.8	3.2–5.3	5.7–9.6	3.9–5.7	8.4–11.7	4.8–9.3	7.4–10.1	3.8–8.8

Error rates for the following scenarios: equally sized trials; one small trial, 1/10th of other trials; 50–50% small and large trials (ratio 1:10); one large trial (10 times larger than other trials). No of trials: number of trials. *DL*: DerSimonian & Laird meta-analysis method. *HKSJ*: Hartung-Knapp-Sidik-Jonkman meta-analysis method.

### Error rates for risk ratio outcomes

The results of the simulations for studies with a risk ratio outcome were quite similar to the error rates for the continuous outcomes, but there was more variation in the error rates: they depended on the group sizes and the risks (from 0.1 to 0.9). For low heterogeneity (I^2^ = 0.25), the DL error rates ranged from 2.2% to 15.5%, whereas the HKSJ rates were slightly better: 2.8–10.6%. However for I^2^ = 0.9 the DL rates ranged from 6.4% to 33.7%, compared to HKSJ rates of 2.7% to 10.2%. When there was no heterogeneity (I^2^ = 0), the DL error rates ranged between 0.9% and 4.3%, and the HKSJ rates between 2.1% and 6.9%. For odds ratios, the results were again quite similar. See Table 1 for a selection of results, and the Additional file 2: Figure S1 and Additional file 3: Figure S2.

### Empirical results for CDSR 2012

Selection of the first meta-analyses in the systematic reviews added in 2012 to the CDSR and based on at least three studies resulted in 689 meta-analyses (255 meta-analyses with a continuous outcome and 434 meta-analyses with a dichotomous outcome).

The continuous outcome meta-analyses were based on a median of five trials (Q1-Q3: 3–9) with a median ratio between the largest and the smallest trial of 5 (Q1-Q3: 3–10). Using the DL method, 130 (51.0%) of the 255 meta-analyses were nominally statistically significant compared to 102 (40.0%) when the HKSJ method was used (Table 2). Of the 130 meta-analyses that were significant with the DL method, 31 (23.8%) were not significant with the HKSJ method, while three meta-analyses were significant with the HKSJ method but not with the DL method. In the selection of meta-analyses based on at most five studies and with large ratios between the study sizes (ratio > 5) 13 (59.1%) of the 22 meta-analyses significant with the DL method were not significant with the HKSJ method and none of the meta-analyses was only significant with the HKSJ method.

**Table 2:** Number (%) of statistically significant Cochrane meta-analyses according to the DerSimonian-Laird and Hartung-Knapp-Sidik-Jonkman methods

**Outcome**	**Selected meta-analyses**	**N**	**DL test significant**	**HKSJ test significant**	**HKSJ test not significant, positive DL test**
Continuous	All	255	130 (51.0)	102 (40.0)	31/130 (23.8)
	Ratio > 5, < = 5 studies	46	22 (47.8)	13 (28.3)	13/22 (59.1)
Dichotomous	All	434	185 (42.6)	147 (33.9)	48/185 (25.9)
	Ratio > 5, < = 5 studies	76	28 (36.8)	15 (19.7)	14/28 (50.0)

*All*: all meta-analyses with a continuous or dichotomous outcome that fulfilled the following criteria: the first meta-analysis in a review in the Cochrane Database for Systematic Reviews Issues of 2012, based on at least three studies. *Ratio >5, < = 5 studies*: a selection of these meta-analyses based on at most five studies, where the ratio of the largest vs. the smallest trial size was > 5. *DL*: DerSimonian & Laird meta-analysis method. *HKSJ*: Hartung-Knapp-Sidik-Jonkman meta-analysis method. *DL test significant*: DL p-value <0.05; *HKSJ test significant*: HKSJ p-value < 0.05. Note that in a few cases the HKSJ test was significant when the DL test was not.

The 434 dichotomous meta-analyses were based on a median of six trials (Q1-Q3: 4–10) with a median ratio between the largest and the smallest trial of 6 (Q1-Q3: 3–16). Of the 434 meta-analyses, 185 (42.6%) were nominally statistically significant with DL and 147 (33.9%) with HKSJ (Table 2). Of the 185 meta-analyses that were significant with the DL method, 48 (25.9%) were not significant with the HKSJ method, while the opposite scenario was seen in 10 cases. In the selection of small meta-analyses with large ratios between the study sizes 14 (50.0%) of the 28 meta-analyses significant with the DL method were not significant with the HKSJ method, while the opposite scenario occurred once.

Summarizing, the DL method resulted in statistically significant results in 315/689 (45.7%) of the meta-analyses; 79 of these 315 “positive” DL results (25.1%) were not significant with the HKSJ method, while the opposite scenario (significant only by HKSJ) was rarely seen (14 meta-analyses). In the selection of small meta-analyses (< = 5 studies) with large ratios between the study sizes (ratio > 5), the difference between the DL and HKSJ results was even larger.

### Easy method for the conversion of DL into HKSJ results

We present two examples to illustrate how DL results can be used to carry out an HKSJ analysis, resulting in HKSJ-confidence intervals and p-values. An Excel file is available as Additional file 1 (web material). The results can also be created with R, Appendix 3.

#### Example 1: conversion to HKSJ for a continuous outcome

The first three columns of Table 3 show the results of a meta-analysis on the effect of zinc for the treatment of a common cold, published in a Cochrane review [24]. The outcome was severity of cold symptoms scoring, and was based on a total of 513 participants. The first column shows the identifiers of the studies, the second column the results *y*_*i*_ of the individual studies and the third column contains the weights *w*_*i*_ from the DL analysis, copied from the review. Only these three columns are needed for the post-hoc calculations.

**Table 3:** Conversion of DerSimonian-Laird results into Hartung-Knapp-Sidik-Jonkman results for a continuous outcome: severity of cold symptoms

**DerSimonian and Laird results**			***Calculations for Hartung-Knapp- Sidik-Jonkman***	
**Study**	**Study results SMD y**_**i**_	**Weights w**_**i**_	***(y***_***i***_ ***–y)***^***2***^	***w***_***i ***_**× *(y***_***i***_ ***–y)***^***2***^
Kurugol 2006a	−0.04	24.0	*0.1225*	*2.94*
Kurugol 2007	−0.07	22.2	*0.1024*	*2.27*
Petrus 1998	−0.31	21.3	*0.0064*	*0.14*
Prasad 2000	−1.36	15.5	*0.9409*	*14.58*
Prasad 2008	−0.54	17.0	*0.0225*	*0.38*
	*y* = −0.39	*Sum: 100.0*		*Sum: 20.31*
5 studies, I^2^ = 75.0%, τ^2^ = 0.13				
DL pooled result [95% CI]: SMD = −0.39 [−0.77, –0.02]; z = 2.05; P–value = 0.04				
HKSJ pooled result [95% CI]: SMD = −0.39 [−1.02, 0.24]; t = 1.73; P–value = 0.16 (df = 4)				

*SMD*: Standardized mean difference. *DL*: DerSimonian & Laird meta-analysis method. *HKSJ*: Hartung-Knapp-Sidik-Jonkman meta-analysis method. *CI*: Confidence Interval, *df*: degrees of freedom, ×: multiplication sign. The pooled effect y and the weights w_i_ originate from the DL random-effects analysis.

The following steps carry out an HKSJ analysis:

1. Determination of the standard error:

a. Based on the overall summary difference *y* = −0.39, calculate the HKSJ factors

*w*_*i*_×*(y*_*i*_*-y)*^*2*^ for each of the studies (see the fifth column for the results).

b. Add the HKSJ factors and divide them by the sum of the weights. This results in 20.31/100 = 0.2031.

c. Divide by *k-1*, whereby *k* is the number of studies. In this situation *k* = 5 and 0.2031/4 = 0.0508. This is the weighted variance of the pooled treatment effect according to the HKSJ approach.

d. Taking the square root leads to the standard error: SE = √0.0508 = 0.225.

2. Determination of the 95% confidence interval (CI):

a. To determine the half-width of the 95% CI, the SE must be multiplied with the 97.5%-quantile of the t-distribution with *k - 1* degrees of freedom. Its value can be obtained through Excel: TINV(0.05, k-1), where *k* is the number of studies. This results in 2.78, so the half-width of the 95% CI is 2.78*0.225 = 0.63. The t-value can also be found on the internet, for example at http://www.danielsoper.com/statcalc3/calc.aspx?id=10.

The quantiles of the t-distribution can be found through statistical packages as well. In SPSS: select ‘compute variable’, function group ‘Inverse DF’, function IDF.T(.975,k-1), or in SAS: tinv(.975,k-1).

b. The HKSJ 95% CI then is *y* ± half-width of the CI, i.e. -0.39 ± 0.63 or [-1.02; 0.24].

3. Determination of the p-value:

a. Calculate the t-statistic: t = *y*/SE = −0.39/0.225 = −1.73. If the result is negative, as in this situation, simply change the sign, so t = 1.73.

b. Determine the corresponding two-sided p-value with Excel: TDIST(1.73,4,2), or with the internet site http://www.danielsoper.com/statcalc3/calc.aspx?id=8. The two-sided P-value according to the HKSJ method then is 0.16.

This p-value can also be obtained through SPSS: ‘compute variable’, function group ‘CDF & noncentral CDF’, function ‘CDF.T’. This yields CDF.T(1.73, 4), similar to SAS, cdf(‘T’, 1.73, 4) = 0.92066. The two-sided HKSJ p-value then is 2×(1–0.92066) ~0.16.

In this example on the efficacy of zinc, based on only five trials and high heterogeneity (I^2^ = 75%), the results of the DL and HKSJ analyses differ substantially.

#### Example 2: conversion to HKSJ for outcomes that require a log transformation

When the outcome of the meta-analysis is a risk ratio (RR), odds ratio (OR), hazard ratio (HR) or Poisson rate, the analysis has to be conducted on the natural logarithm (ln) of the treatment effect. In all other aspects the procedure is exactly the same as for a continuous outcome. As an example we show the overall survival for post-remission therapy for adult acute lymphoblastic leukemia, comparing patients with and without a donor, as presented in a Cochrane Review [25]. The first three columns of Table 4 show the results of a meta-analysis with the HR as outcome.

**Table 4:** Conversion of DerSimonian-Laird results into Hartung-Knapp-Sidik-Jonkman results for a logarithm based outcome: hazard ratios

**DerSimonian and Laird results**			***Calculations for Hartung-Knapp-Sidik Jonkman***		
**Study**	**Study results HR y**_**i**_	**Weights w**_**i**_	***ln(y***_***i***_ ***)***	***(ln(y***_***i ***_ ***)–ln(y))***^***2***^	***w***_***i***_**×** ***(ln(y***_***i ***_ ***)–ln(y))***^***2***^
Cornelissen 2009	0.81	5.0	*−0.21*	*0.00*	*0.02*
De Witte 1994	0.67	2.1	*−0.40*	*0.06*	*0.13*
Fielding 2009	0.80	11.5	*−0.22*	*0.01*	*0.06*
Goldstone 2008	0.91	46.7	*−0.09*	*0.00*	*0.15*
Hunault 2004	0.56	2.9	*−0.58*	*0.18*	*0.53*
Labar 2004	0.98	9.3	*−0.02*	*0.02*	*0.16*
Ribera 2005	1.24	3.9	*0.22*	*0.13*	*0.52*
Sebban 1994	0.75	12.7	*−0.29*	*0.02*	*0.24*
Takeuchi 2002	0.95	3.9	*−0.05*	*0.01*	*0.04*
Ueda 1998	0.66	2.0	*−0.42*	*0.07*	*0.14*
	y = 0.86	*Sum: 100.0*			*Sum: 1.99*
10 studies, I^2^ = 0.0, τ^2^ = 0.0.					
DL pooled result [95% CI]: HR = 0.86 [0.77, 0.97]; z = −2.48; P–value = 0.013.					
HKSJ pooled result [95% CI]: HR = 0.86 [0.77, 0.96]; t = −3.19; P–value = 0.011 (df = 9).					

*HR*: Hazard Ratio for donor versus no-donor; *ln*: natural logarithm; *DL*: DerSimonian & Laird meta-analysis method. *HKSJ*: Hartung-Knapp-Sidik-Jonkman meta-analysis method. *CI*: Confidence Interval, *df*: degrees of freedom, ×: multiplication sign. The pooled effect y and the weights w_i_ originate from the DL random-effects analysis on log scale.

1. Determination of the standard error:

a. Calculate the natural logarithm of the pooled estimate: ln(*y*) = ln(0.86) = −0.15. Calculate the natural logarithms of the study outcomes (column 4) and use these to calculate the HKSJ factors *w*_*i*_×*(ln(y*_*i*_*)-ln(y))*^*2*^ for each of the studies (column 6).

b. Add the HKSJ factors and divide them by the sum of the weights. This leads to 1.99/100 = 0.0199.

c. As there are 10 studies, divide by *k-1* = 9: 0.0199/9 = 0.0022.

d. Taking the square root leads to the standard error: SE = √0.0022 = 0.047.

2. Determination of the 95% CI:

a. On the ln scale, the half-width of the 95% CI is TINV(0.05, 9) × 0.047 = 2.26 × 0.047 = 0.106 (Excel).

b. The 95% CI for the ln HR is −0.15 ± 0.106, i.e. [−0.26; -0.04].

c. The HKSJ 95% CI for the HR is [e^-0.26^; e^-0.04^], i.e. [0.77; 0.96].

3. Determination of the p-value:

a. Calculate the t-statistic: t = ln(y)/SE = −0.15/0.047 = −3.19. Neglecting the negative sign, we obtain t = 3.19.

b. Use Excel, Internet or a statistical package to calculate the two-sided p-value according to the HKSJ method, see Example 1. Excel: p-value = TDIST(3.19,9,2) = 0.011; SPSS: CDF.T(3.19, 9) = 0.995, so that the p-value is 2×(1–0.995) = 0.011.

In this example, results of the DL and HKSJ analyses hardly differ.

## Discussion

The DL approach to random effects meta-analysis is still the standard method, almost to the exclusion of all other methods. This might be considered remarkable, bearing in mind the high false positive rates of the DL method which have been shown repeatedly with simulations [4-14] and also an empirical study suggesting that results are sensitive to the choice of random effects analysis method [26]. Thorlund et al. did an empirical assessment in 920 Cochrane primary outcome meta-analyses of > = 3 studies of method-related discrepancies [26]. In total, 326 (35.4%) meta-analyses were statistically significant when the analysis was based on a t-distribution – as in the HKSJ method – and 414 (45%) when it was based on the normal distribution as in the DL method. Our evaluation of Cochrane meta-analyses of interventions resulted in a similar result: a substantially larger amount of significant findings with the DL method than with the HKSJ method. Our simulations suggest that among the DL significant findings in the Cochrane reviews there may be a considerable number of false positives.

DL results can easily be converted into HKSJ results, which have a much better performance. We confirmed this with simulations, for mixtures of trial size distributions in settings with up to 20 trials per meta-analysis. When there was heterogeneity, the mean error rates of the DL approach were consistently higher than those of the HKSJ approach, although also the latter doubled to 10% in scenarios with only one large trial. When there was no heterogeneity, the DL error rates were lower than 5%, and the HKSJ rates were approximately 5%.

However, there are some limitations with respect to the HKSJ analysis method. Although the error rates of the HKSJ method were closer to the 5% level than those of the DL method, our simulations showed that in some scenarios the HKSJ error rates more or less doubled, although the DL error rates could be more than four times too high in these same settings. Hence, the results of the HKSJ analysis are also not perfect. Like we hypothesized, the error rates were maximal if one of the trials in the meta-analysis was substantially larger than the other ones.

Further, when study numbers are small, the distribution of the treatment effects is unknown and does not necessarily follow the normal or t-distribution. Kontopantelis and Reeves [27] showed that with slight heterogeneity the coverage of the HKSJ method was consistently 94% when the true effects were not distributed according to the normal or t-distribution, but with larger heterogeneity the non-parametric permutation (PE) method of Follmann and Proschan [7] performed better than the HKSJ method. However, the PE method can only be performed when the number of studies is larger than five, whereas many meta-analyses are smaller [15]. Several other methods have been developed, like the Quantile Approximation (QA) method [28], the Profile Likelihood approach [29], natural weighting instead of empirically based weighting of studies [30], use of fixed effects estimates with a random effects approach to heterogeneity [31] and more recently, higher-order likelihood inference methods [32]. However, most of these methods are based on asymptotic statistics and they may therefore be less robust in case of a limited number of trials, or they remain difficult to use in practice, because no statistical packages are available to perform them and it is very difficult to carry out the calculations with standard software. Regarding the non-asymptotic, computationally straightforward QA method, Sánchez-Meca and Marín-Martínez [13] have already shown that it was outperformed by the HKSJ method. It would require a very extensive evaluation to investigate the performance of all of these methods. We restricted ourselves to the HKSJ method, because of its computational simplicity and we show that HKSJ results can easily be derived from DL results.

As far as we know, we are the first to present systematically the error rates in relation to explicit trial size mixtures when the numbers of trials range from 2 to 20. Follmann and Proschan [7] show that for certain trial size mixtures and low numbers of trials the DL error rates can be highly increased, however, they did not evaluate the HKSJ method. The results reported by Hartung, Knapp and Makambi [4-6,8,9] imply that for meta-analyses of three, six or twelve studies the DL error rates for studies with similar sizes were closer to 5% than for studies of different sizes, and that the HKSJ method performed much better than DL in the latter situation. However they did not report the explicit relationship between the trial size mixtures and error rates as we do (Table 1). Sánchez-Meca and Marín-Martínez [13] also varied the sample size ratios in their simulations. They concluded that the average sample size scarcely affected the performance of the different methods, but this was based on the combined results of 5–100 studies and they presented no results of particular trial size mixtures.

As all studies show that in settings with few studies the HKSJ method always resulted in error rates closer to 5% than the DL method, the latter method should not be used and the HKSJ method should be the standard approach. To facilitate its more widespread application, the conversion of DL results into HKSJ results is presented step by step. At the same time, we urge caution when any random effects model, including HKSJ, is applied to situations where there are very few studies, and even more so when the sample sizes of the combined studies are very different. Even the HKSJ confidence intervals may be conservatively narrow in these situations and inferences may be spurious, if the confidence intervals are taken at face value.

## Conclusions

Our simulations showed that the HKSJ method for random effects meta-analysis consistently results in more adequate error rates than the common DL method, especially when the number of studies is small. The HKSJ method can easily be applied routinely in meta-analyses. However, even with the HKSJ method, extra caution is needed when there are = < 5 studies of very unequal sizes.

## Appendix 1: Statistical details

### Random effects meta-analysis model

For *k* studies, let the random variable y_i_ be the effect size estimate from the *i*^th^ study. The random effect model can be defined as follows:

$$y_{i}=\delta_{i}+e_{i}$$

for *i =1, . . ., k,* where *δ*_*i*_ = *δ* + *d*_*i*_; *e*_*i*_ and *d*_*i*_ independent, $e_{i}\sim N\left(0,\;\in_{i}^{2}\right)$ and *d*_*i*_ ~ *N*(0, *τ*^2^).$\in_{i}^{2}$ is the within-study variance, describing the extent of estimation error of *δ*_*i*_, and the parameter *τ*^*2*^ represents the heterogeneity of the effect size between the studies.

For studies with dichotomous outcomes where no events were observed in one or both arms, the computation of the random effects model yields a computational error. In these cases, before performing any meta-analysis, we added 0.5 to all cells of such a study.

### Random effects analysis

Let *w*_*i*_ be the fixed effects weights, i.e. the inverse of the within-study variance ${\hat{\in }}_{i}^{2}$, and let ${\hat{y}}_{F}$ be the fixed effects estimate of δ.

Let *Q* be the heterogeneity statistic $Q=\sum w_{i}{\left(y_{i}-{\hat{y}}_{F}\right)}^{2}$.Then

$${\hat{\tau }}^{2}=\max\left(0,\frac{Q-\left(k-1\right)}{\sum w_{i}-\sum w_{i}^{2}/\sum w_{i}}\right)$$

is an estimate of the variance τ^2^.

The random effects estimate for the average effect size δ is

$${\hat{y}}_{R}=\frac{\sum w_{i}^{\tau }y_{i}}{\sum w_{i}^{\tau }}$$

where

$$w_{i}^{\tau }=\frac{1}{{\hat{\in }}_{i}^{2}+\;{\hat{\tau }}^{2}}.$$

The DerSimonian and Laird method estimates the variance of ${\hat{y}}_{r}$ by

$${\text{var}}_{\mathrm{DL}}=\frac{1}{\sum w_{i}^{\tau }}$$

and uses the normal distribution to derive P-values and confidence intervals.

In contrast, the Hartung, Knapp, Sidik and Jonkman method estimates the variance of ${\hat{y}}_{r}$ by

$${\mathrm{var}}_{\mathrm{HKSJ}}=\frac{\sum w_{i}^{\tau }{\left(y_{i}-{\hat{y}}_{r}\right)}^{2}}{\left(k-1\right)\sum w_{i}^{\tau }}\;$$

and uses the t-distribution with k-1 degrees of freedom to derive P-values and confidence intervals, with *k* the number of studies in the meta-analysis.

### Heterogeneity estimates

Although ${\hat{\tau }}^{2}$ or *Q* can be used as measures of the heterogeneity, Higgins and Thompson [16] propose

$$I^{2}=\frac{Q-\left(k-1\right)}{Q}$$

I^2^ is a relative measure. It compares the variation due to heterogeneity (*τ*^*2*^) to the total amount of variation in a ‘typical’ study (*τ*^*2*^ +*∈*^*2*^), where *∈* is the standard error of a typical study of the review [33]:

(1) $$I^{2}=\;\frac{\tau^{2}}{\tau^{2}+\epsilon^{2}}$$

## Appendix 2: The simulations

### The parameters in the scenarios for the simulations

– The number of trials per series *k* = 2 – 20;

– The average group size in a series of trials: 25, 50, 100, 250, 500 or 1000 subjects per group per trial;

– The trial size mixtures: we simulated series with 25, 50 or 75% large trials, series with exactly one large or one small trial, and series where all trials were of equal size;

– The ratio of the study sizes: for the series with small and large studies, the large study was 10 times the size of a small study.

The simulations were programmed in SAS, version 9.2. The scenarios were evaluated 10,000 times, for heterogeneity levels I^2^ = 0, 0.25, 0.5, 0.75, and 0.9, and at a nominal significance level α = 0.05 (two-sided).

#### A. The simulation for normally distributed outcomes

1. For each scenario, and each value of *I*^*2*^, we used eq. (1) to calculate the variance τ^2^. So

(2) $$\tau^{2}=\epsilon^{2}\;\frac{I^{2}}{1-I^{2}}$$

where $\epsilon^{2}=\frac{1}{k}\;\sum \frac{2\;\sigma^{2}}{n_{i}}$, with *n*_*i*_ the groupsize of trial *i (i = 1…k)* and σ the standard deviation of the outcome variable of the trials. As *σ* is only a scaling factor and the results only depend on the ratio *τ/σ*, we have set *σ* = 1 in the simulations.

2. For each trial i:

a. We determined the ‘true’ trial effect size δ_i_, where δ_i_ was a random draw from the normal distribution with mean 0 and variance *τ*^*2*^.

b. We generated the trial outcome based on a normal distribution with mean δ_i_ and variance 2*σ*^2^/*n*_*i*_ =  2/*n*_*i*_.

c. We generated the variance of the trial outcome based on a χ^2^ distribution with 2n_i_-2 degrees of freedom, divided by n_i_-1.

3. For each series:

A DerSimonian and Laird analysis and an HKSJ analysis were carried out.

4. For each scenario*, I*^*2*^ and each meta-analysis method, we calculated the error rate, i.e. the percentage of series that had a statistically significant (p<0.05) outcome.

#### B. The simulations for the odds ratio

1. When the outcome was dichotomous, we had to choose an additional parameter: the overall event rate *p*_*0*_. We varied the *p*_*0*_ between 0.1 and 0.9 and for each value we used (2) to calculate *τ*^*2*^, with

$$\varepsilon^{2}=\frac{1}{k}\;\sum \frac{1}{n_{i}\;}\;\left(\frac{2}{p_{0}}+\frac{2}{1-p_{0}}\right)$$

2. For each trial i:

a. We determined the ‘true’ trial effect size ln(odds ratio_i_) = δ_i_, where δ_i_ was a random draw from the normal distribution with mean 0 and variance τ^2^.

b. We calculated the event rates *p*_*a*_ and *p*_*b*_ in the two groups, such that: ln(p_a_ /(1-p_a_)) = ln(p_0_ /(1-p_0_)) - δ_i_/2, and ln(p_b_ /(1-p_b_)) = ln(p_0_ /(1-p_0_)) + δ_i_/2.

c. We generated the observed event rates *P*_*a*_ and *P*_*b*_ in each group based on Bernouilli distributions with event rates *p*_*a*_ and *p*_*b*_, respectively.

d. Based on *P*_*a*_ and *P*_*b*_, we calculated the natural log of the odds ratio and its variance (1/*P*_*a*_ +1/(1- *P*_*a*_) +1/*P*_*b*_ +1/(1- *P*_*b*_))/*n*_*i*_.

Steps 3 and 4 were the same as for a continuous outcome.

#### C. The simulations for the risk ratio

The risk ratio simulation was similar to the odds ratio simulation, but the variance was different:

$$\varepsilon^{2}=\frac{1}{k}\;\sum \frac{1\;}{n_{i}}\;\left(\frac{2}{p_{0}}\;-\;2\right).$$

Furthermore, for each trial:

a. We determined the ‘true’ trial risk ratio ln(risk ratio_i_) = δ_i_ , where δ_i_ was a random draw from the normal distribution with mean 0 and variance τ^2^.

b. We calculated the event rates *p*_*a*_ and *p*_*b*_ in the two groups, such that:

ln(p_a_) = ln(p_0_) - δ_i_/2 and ln(p_b_) = ln(p_0_) + δ_i_/2. Event rates below 0.01 or above 0.99 were replaced by 0.01 or 0.99, respectively.

c. We generated the observed event rates *P*_*a*_ and *P*_*b*_ in each group based on Bernouilli distributions with event rates *p*_*a*_ and *p*_*b*_, respectively.

d. This led to the natural log of the risk ratio and its variance (1/*P*_*a*_ +1/*P*_*b*_ - 2)/*n*_*i*_.

## Appendix 3: R code for the conversion of DL to HKSJ results

The R package metafor [20] can also be used to perform an HKSJ analysis. The implementation is based on the meta-regression paper by Knapp and Hartung [34]: when no covariates or moderator variables are used, the meta-regression reduces to a random effects meta-analysis as proposed by Hartung/Knapp and Sidik/Jonkman.

The usual approach to perform an HKSJ analysis with metafor is based on study effects combined with fixed effects weights or standard errors. In our examples the HKSJ method must be applied on random effects weights instead of fixed effects weights. This can be done by choosing a fixed effects analysis (method=“FE”) in combination with the HKSJ method. This will result in warnings, because in general the HKSJ adjustment is not meant to be used in combination with a fixed effects analysis. In this case, the warnings can be neglected. The code is kindly provided by G Knapp.

### Code for HKSJ conversion in R

library(metafor)

#### First example

y <− c(−0.04, -0.07, -0.31, -1.36, -0.54)

w <− c( 24.0, 22.2, 21.3, 15.5, 17.0)

rma.uni(y, vi = 1/w, method="FE", knha=TRUE)

Output is presented in Table 5.

**Table 5:** R output for first example (Hartung-Knapp-Sidik-Jonkman method)

	**Estimate**	**SE**	**t-value**	**p-value**	**CI.LB**	**CI.UB**
**R output**	−0.3938	0.2254	−1.7473	0.1555	−1.0195	0.2319

Relevant part from output from R package metafor. *SE*: standard error; *CI.LB*: lower bound of 95% confidence interval; *CI.UB*: upper bound of 95% confidence interval.

#### Second example (ln HR)

y <− c(0.81, 0.67, 0.80, 0.91, 0.56, 0.98, 1.24, 0.75, 0.95, 0.66)

w <− c( 5.0, 2.1, 11.5, 46.7, 2.9, 9.3, 3.9, 12.7, 3.9, 2.0)

# meta-analysis on log scale (ln HR). Note the brackets around the following syntax!

(hr <− rma.uni(log(y), vi=1/w, method="FE", knha=TRUE))

# backtransformation:

exp(hr$b)

exp(c(hr$ci.lb, hr$ci.ub)) (Table 6).

**Table 6:** R output for second example (Hartung-Knapp-Sidik-Jonkman method)

	**Estimate (HR)**	**SE**	**t-value**	**p-value**	**CI.LB**	**CI.UB**
**R output**	−0.1458	0.0470	−3.1031	0.0127	−0.2521	−0.0395
**After back-transformation**	0.8643				0.7772	0.9613

Relevant part from output from R package metafor. *HR*: hazard ratio; *SE*: standard error; *CI.LB*: lower bound of 95% confidence interval; *CI.UB*: upper bound of 95% confidence interval.

## Competing interests

The authors declare that they have no competing interests.

## Authors’ contributions

GB conceived the idea. JIH contributed substantially to the study design, developed the software and performed the statistical analyses. JIH, GB and JPAI drafted the manuscript, and read and approved the final manuscript.

## Pre-publication history

The pre-publication history for this paper can be accessed here:

http://www.biomedcentral.com/1471-2288/14/25/prepub

## Supplementary Material

Additional file 1 Excel template for the conversion of DL to HKSJ results (Web material). Click here for file

Additional file 2: Figure S1 DerSimonian-Laird and Hartung-Knapp-Sidik-Jonkman error rates for Risk Ratios, for various I^2^ and mixtures of trial sizes. A: Equally sized trials; B: One small trial, 1/10^th^ of other trials; C: 50–50 small and large trials (ratio 1:10); D: one large trial (10 times larger than other trials). Vertical bars refer to the minimum and maximum error rates over the group sizes. The lines connect the means of these error rates. DL: DerSimonian & Laird meta-analysis method. SJ: Hartung-Knapp-Sidik-Jonkman meta-analysis method. Click here for file

Additional file 3: Figure S2 DerSimonian-Laird and Hartung-Knapp-Sidik-Jonkman error rates for Odds Ratios, for various I^2^ and mixtures of trial sizes. A: Equally sized trials; B: One small trial, 1/10^th^ of other trials; C: 50–50 small and large trials (ratio 1:10); D: one large trial (10 times larger than other trials). Vertical bars refer to the minimum and maximum error rates over the group sizes. The lines connect the means of these error rates. DL: DerSimonian & Laird meta-analysis method. HKSJ: Hartung-Knapp-Sidik-Jonkman meta-analysis method. Click here for file
