# Supplementary material for: The Hartung-Knapp-Sidik-Jonkman method for random effects meta-analysis is straightforward and considerably outperforms the standard DerSimonian-Laird method
Source: BMC Med Res Methodol. 2014 Feb 18;14:25. doi: 10.1186/1471-2288-14-25 (PMC4015721; doi:10.1186/1471-2288-14-25)
Supplement: Additional file 2: Figure S1 — DerSimonian-Laird and Hartung-Knapp-Sidik-Jonkman error rates for Risk Ratios, for various I2 and mixtures of trial sizes. A: Equally sized trials; B: One small trial, 1/10th of other trials; C: 50–50 small and large trials (ratio 1:10); D: one large trial (10 times larger than other trials). Vertical bars refer to the minimum and maximum error rates over the group sizes. The lines connect the means of these error rates. DL: DerSimonian & Laird meta-analysis method. SJ: Hartung-Knapp-Sidik-Jonkman meta-analysis method. [file 1471-2288-14-25-S2.docx]

**















**

**Figure Web.1** DerSimonian-Laird and Hartung-Knapp-Sidik-Jonkman error rates for **Risk Ratios**, for various I^2^ and mixtures of trial sizes.

**Legend:**

A: Equally sized trials; B: One small trial, 1/10th of other trials; C: 50–50 small and large trials (ratio 1:10); D: one large trial (10 times larger than other trials). Vertical bars refer to the minimum and maximum error rates over the group sizes. The lines connect the means of these error rates. DL: DerSimonian & Laird meta-analysis method. SJ: Hartung-Knapp-Sidik-Jonkman meta-analysis method.
